# Association between Arterial Stiffness and Serum L-Octanoylcarnitine and Lactosylceramide in Overweight Middle-Aged Subjects: 3-Year Follow-Up Study

**DOI:** 10.1371/journal.pone.0119519

**Published:** 2015-03-17

**Authors:** Minjoo Kim, Saem Jung, Sang-Hyun Lee, Jong Ho Lee

**Affiliations:** 1 National Leading Research Laboratory of Clinical Nutrigenetics/Nutrigenomics, Department of Food and Nutrition, College of Human Ecology, Yonsei University, Seoul, Korea; 2 Department of Food and Nutrition, Brain Korea 21 PLUS Project, College of Human Ecology, Yonsei University, Seoul, Korea; 3 Department of Family Practice, National Health Insurance Corporation Ilsan Hospital, Goyang, Korea; 4 Yonsei University Research Institute of Science for Aging, Yonsei University, Seoul, Korea; Vanderbilt University, UNITED STATES

## Abstract

Existing data on the association between being overweight and cardiovascular morbidity and mortality risk in adults are inconsistent. We prospectively and longitudinally investigated the effects of weight on arterial stiffness and plasma metabolites in middle-aged subjects (aged 40–55 years). A group of 59 individuals who remained within the range of overweight during repeated measurements over a 3-year period was compared with a control group of 59 normal weight subjects who were matched for age and gender. Changes in metabolites by UPLC-LTQ-Orbitrap mass spectrometry and changes in brachial-ankle pulse wave velocity (ba-PWV) were examined. At baseline, the overweight group showed higher BMI, waist circumference, triglyceride, free fatty acid (FFA), glucose, insulin, and hs-CRP, and lower HDL-cholesterol than controls. After 3 years, the changes in waist circumference, diastolic and systolic blood pressure (DBP and SBP), triglyceride, FFA, glucose, insulin, hs-CRP, and ba-PWV observed in the overweight group were significantly different from those in the control group after adjusting for baseline levels. Furthermore, the overweight group showed greater increases in L-octanoylcarnitine (*q*=0.006) and decanoylcarnitine (*q*=0.007), and higher peak intensities of L-leucine, L-octanoylcarnitine, and decanoylcarnitine. Multiple linear regression analysis showed that the change in ba-PWV was independently and positively associated with changes in L-octanoylcarnitine, lactosylceramide, and SBP, and with baseline BMI. Our results indicate that the duration of overweight is an important aggravating factor for arterial stiffness, especially during middle age. Additionally, an age-related increase in plasma L-octanoylcarnitine, lactosylceramide, SBP, and baseline BMI are independent predictors of increased arterial stiffness in middle-aged individuals.

## Introduction

A recent systematic review of studies examining the relationship between standard body mass index (BMI) categories and all-cause mortality found that obesity was associated with higher mortality risk whereas being overweight was associated with a lower risk of mortality compared with normal weight [1]. However, Borrell et al. [2] did not find a protective effect for overweight adults with respect to all-cause or cardiovascular disease (CVD)-specific mortality risk. In addition, McDonough et al. [3] showed that overweight individuals were 20% more likely to have a lower health-related quality of life than people of normal weight. These inconsistent results for overweight individuals could result from differences between ethnic groups or the wide age range covered in previous studies.

It is well established that greater vascular stiffness is associated with increased CVD risk in adults [4]. Arterial stiffness, measured as central pulse wave velocity (PWV), is most strongly correlated with CVD [5] and is predictive of both cardiovascular morbidity and all-cause mortality in adults [6]. PWV was shown to be associated with metabolic syndrome even in the absence of hypertension [7]. In addition, several studies in healthy individuals demonstrated parallel increases in central and brachial systolic blood pressure and arterial stiffness as BMI increases [8–10]. Moreover, a recent follow-up study with middle-aged subjects revealed that the age-related increase in arterial stiffness is greater in the presence of metabolic syndrome with increased BMI and waist circumference [11]. Therefore, we hypothesized that individuals, who remain overweight over time compared to those who remain normal weight, will demonstrate greater increases in metabolic abnormalities and arterial stiffness not only with aging also longer duration of overweight, compared with individuals. To investigate the effects of overweight on metabolic changes and arterial stiffness, we examined changes in arterial stiffness, measured as brachial-ankle PWV (ba-PWV) which correlates with both central and peripheral PWVs [12]. Metabonomics is a sensitive and powerful tool for biomarker discovery and understanding disease pathophysiology [13,14]. Although metabonomics is a potentially useful approach for the exploration of arterial stiffness, metabolic variation and the duration of overweight during aging have not been clarified. Therefore, we performed metabolic profiling by UPLC-LTQ-Orbitrap mass spectrometry in overweight middle-aged (40-to 55-year-old) individuals and normal weight controls over a 3-year period.

## Materials and Methods

### Subjects

This study included 118 individuals (aged 40–55 years) who underwent triennial medical evaluation at the National Health Insurance Corporation (NHIC) Ilsan Hospital in Goyang, Korea, during the period from January 2009 to February 2012. We identified a group of 59 individuals who remained within the range of overweight (25 ≤ BMI <30 kg/m^2^) during repeated measurements over a 3-year period. This overweight group was compared with a control group of 59 normal weight subjects (18.5 ≤ BMI <25 kg/m^2^) who were matched for age and gender. Subjects were excluded if they had any diagnosis of vascular disease, cancer (clinically or by anamnesis), renal disease, liver disease, thyroid disease, chronic alcoholism, and acute or chronic inflammatory diseases; took antioxidant; or were taking glucose-lowering, lipid-lowering or antihypertensive medications. The purpose of the study was carefully explained to all participants, and written consent was obtained prior to their participation. The Institutional Review Board of the NHIC-sponsored Ilsan Hospital and Yonsei University provided ethical approval of the study protocol, which was performed according to the Helsinki Declaration.

### Anthropometric parameters, blood pressure, and blood collection

Body weight (UM0703581, Tanita, Tokyo, Japan) and height (GL-150, G-tech international, Uijeongbu, Korea) of unclothed subjects without shoes were measured in the morning for calculation of body mass index (BMI, kg/m^2^). Waist circumference was measured at the umbilical level with the subjects standing after normal expiration; hip girth was measured at the widest part of the hip; and the waist to hip ratio (WHR) was calculated. Waist and hip circumferences were measured to the nearest 0.1 cm using a plastic measuring tape. Blood pressure (BP) was measured in the left arm of seated patients with an automatic BP monitor (TM-2654, A&D, Tokyo, Japan) after a 20-min rest. After a 12-hour fasting period, avoid 12-hours of intake meal including alcohol and caffeine consumption, venous blood specimens were collected in EDTA-treated and plain tubes and centrifuged to yield plasma or serum, which were stored at -70°C until analysis.

### Dietary intake assessments and physical activity

Dietary intake was measured using a semi-quantitative food frequency questionnaire (FFQ) and a 24-h recall method. Participants were asked to indicate how frequently, on average, they consumed particular food items through FFQ. Categories were cereals, sugars, protein, fats, vegetables, dairy, fruits, and others and each categories were more specifically divided into several food items. Korean meals are comprised of steamed rice and soup with a variety of side dishes. Rice is the main source of carbohydrate in Korea. Koreans consume rice two or three times a day as a staple food and it supplies 37.9% of their total energy consumption. White rice is usually cooked alone or together with beans and multi-grains in Korea [15]. For the side dishes, people eat Kimchi (traditionally fermented cabbage) at every meal, add various cooked vegetables and sometimes meat or fish. Most of subjects had traditional Korean eating pattern which is consist of steamed rice and soup with some side dishes, three times a day. In addition, we used a 24-h recall method to evaluated daily food intake more precisely; 2 week-days and 1 weekend day. To minimize seasonal differences in dietary intake, we matched each subject among subjects enrolled in a same month. Nutrient intake was calculated as a mean value from a three-day based on the result of 24-h recall method using CAN-pro 3.0. CAN-pro 3.0 is commonly used nutrient analysis software in Korea, reflecting typical ingredients and amounts for Korean diet and it is based on standard tables of food composition from Dietary Reference Intakes for Koreans [16].

Physical activity was assessed with the dietary intakes at baseline and 3-year follow-up. Physical activity was assessed from activity patterns; a mean value of 2 week-days and 1 weekend day, and each item was given activity coefficient considering exercise intensity as follows: Sleeping(0.9), Cleaning(2.7), Cooking(2.6), Dish washing(1.7), Personal(1.5), Driving(1.4), Eating(3.1), Shopping(3.2), Laundry(1.6), Sitting(2), Standing(3.1), Stair(3.5), Walking slow(4.5), and Walking fast(7). Moreover, we also examined type and frequency of regular exercise to consider extra physical activity besides daily life, such as Running(7), Swimming(7), Mountain climbing(7), Tennis(7), and etc. A mean value of activity coefficient were calculated from multiplying time consumed of each item and dividing by 1440 minute. Total energy expenditure was calculated by multiplying activity coefficient by basal energy expenditure from Harris-Benedict equation [17].

### Fasting glucose, insulin, and homeostasis-model assessment of insulin resistance

Fasting glucose levels were analyzed by the hexokinase method using a Hitachi 7600 Autoanalyzer (Hitachi Ltd., Tokyo, Japan). Insulin levels were measured by radioimmunoassay using commercial kits from Immuno Nucleo Corporation (Stillwater, MN, USA). Insulin resistance (IR) was calculated by the homeostasis-model assessment (HOMA) using the following equation: HOMA—IR = [Fasting insulin (μIU/mL) × Fasting glucose (mmol/L)] / 22.5.

### Serum lipid profile and free fatty acids

Fasting levels of total cholesterol and triglyceride were analyzed by enzymatic assay and measured using a Hitachi 7600 Autoanalyzer (Hitachi Ltd., Tokyo, Japan). ApoB-containing lipoproteins were precipitated with dextran-sulfate magnesium, and HDL-cholesterol concentrations in the supernatants were measured enzymatically. For subjects with serum triglyceride levels <400 mg/dL, LDL-cholesterol concentrations were estimated indirectly using the Friedwald formula: LDL-cholesterol = Total-cholesterol—[HDL-cholesterol + (Triglycerides/5)]. For subjects with serum triglyceride levels ≥400 mg/dL, LDL-cholesterol concentrations were measured directly using Hitachi 7600 Autoanalyzer. Free fatty acids (FFA) were analyzed by enzymatic assay [acylCoA synthetase-acylCoA oxidase (ACS-ACOD) method] with a Hitachi 7600 Autoanalyzer.

### Assessment of serum high-sensitivity C-reactive protein and measurement of brachial-ankle pulse wave velocity

The concentration of serum high-sensitivity C-reactive protein (hs-CRP) was measured with an Express Plus TM auto-analyzer (Chiron Diagnostics Co., Walpole, MA, USA) using a commercially available, high-sensitivity CRP-Latex(II) X2 kit (Seiken Laboratories Ltd., Tokyo, Japan). baPWV was measured using an automatic waveform analyzer (Model VP-1000; Nippon Colin Ltd., Komaki, Japan). Subjects were examined in the supine position after 5min of bed rest. Electrocardiogram electrodes were placed on both wrists and a microphone for the phonogram was placed on the left edge of the sternum. Pneumonic cuffs were wrapped around both upper arms and ankles and connected to a plethysmographic sensor to determine the volume pulse waveform. Waveforms for the upper arm (brachial artery) and ankle (tibial artery) were stored for 10-s sample times with automatic gain analysis and quality adjustment. An oscillometric pressure sensor was attached to the cuffs to measure blood pressure at the four extremities. The baPWVs were recorded using a semiconductor pressure sensor (1200 Hz sample acquisition frequency) and calculated using the equation: (La-Lb)/ΔTba. La and Lb were defined as the distance from the aortic valve to the elbow and to the ankle, respectively. The distance from the suprasternal notch to the elbow (La) and from the suprasternal notch to the ankle (Lb) were expressed by: La = [0.2195×height of subject (cm)]-2.0734 and Lb = [0.8129×height of subject (cm)] + 12.328. The time interval between arm and ankle distance (ΔTba) was defined as the pulse transit time between brachial and tibial arterial pressure waveforms. The average baPWVs from both left and right sides, repeated two times, was used for the analysis.

### Global (nontargeted) metabolic profiling of plasma

#### Sample preparation and analysis

Prior to analysis, 800 μL of 80% acetonitrile was added to 100 μL of plasma and the mixture was vortexed and centrifuged at 10,000 rpm for 5 min at 4°C. The supernatant was dried with N_2_, dissolved in 10% methanol, mixed by vortexing, and centrifuged at 10,000 rpm for 5 min at 4°C. The resultant supernatant was transferred into a vial for analysis.

#### Ultra performance liquid chromatography (UPLC)

Plasma extract samples (7 μL) were injected into an Acquity UPLC-BEH-C18 column (2.1 × 50 mm, 1.7 μm; Waters, Milford, MA) that was coupled in-line with a UPLC-LTQ-Orbitrap XL (Thermo Fisher Scientific, USA). The injected samples were equilibrated with water containing 0.1% formic acid. Samples were eluted with an acetonitrile gradient containing 0.1% formic acid at a flow rate of 0.35 mL/min for 20 min. Metabolites were separated by UPLC (Waters, Milford, MA), analyzed, and assigned by LTQ-Orbitrap-XL (Thermo Fisher Scientific, USA). The mass spectrometer was operated in ESI-positive mode. The spray voltage was 5 kV. The flow rate nitrogen sheath gas and the auxiliary gas were 50 and 5 (arbitrary units). The capillary voltage (V), tube-lens voltage (V), and capillary temperature (°C) were kept constant at 35 V, 80 V, and 370°C, respectively. The Orbitrap data were collected in the range of *m*/*z* 50–1,000. For quality control, a mixture of four standard compounds (acetaminophen, sulfadimethoxine, terfenadine, and reserpine) was injected into every 10th sample. The MS/MS spectra of metabolites were obtained by a collision-energy ramp from 55–65 eV, and analyzed with Xcalibur 2.1 and MS Frontier software (Thermo Fisher Scientific, USA).

#### Data processing and identification of metabolites

All MS data including retention times, *m*/*z*, and ion intensities were extracted by SIEVE software (Thermo Fisher Scientific, USA) incorporated into the instrument and assembled into a matrix. SIEVE parameters were set as follows: *m*/*z* range 50–1 000; *m*/*z* width 0.02; retention time width 2.5; and *m*/*z* tolerance 0.005. Metabolites were searched using the following databases: ChemSpider (www.chemspider.com), Human Metabolome (www.hmdb.ca), Lipid MAPS (www.lipidmaps.org), KEGG (www.genome.jp/kegg), and MassBank (www.massbank.jp). Selected metabolites were confirmed using standard samples on the basis of both retention times and mass spectra.

### Statistical analyses

Statistical analyses were performed using SPSS v. 21.0 (IBM SPSS Statistics 21, Chicago, IL, USA). Skewed variables were logarithmically transformed for statistical analyses. For descriptive purposes, mean values are presented using untransformed values. Results are expressed as means ± standard error (SE). A two-tailed *P*-value of <0.05 was considered statistically significant. Differences in clinical variables between control and overweight groups at baseline and 3-year follow-up were tested using Student’s independent *t*-tests. General linear model (GLM) tests were applied to compare changes in variables between the two groups by adjusting for baseline value. Paired *t*-tests were used to evaluate differences between baseline and 3-year follow-up levels in each group. Pearson’s and partial correlation coefficients were used to examine the relationships between variables over time. Multiple regression analyses were performed to identify major plasma metabolites which were associated with blood pressure. False Discovery Rate (FDR) corrected *q*-values were computed using the R package ‘fdrtool’.

Multivariate statistical analysis was performed using SIMCA-P+ software version 12.0 (Umetrics, Umeå, Sweden). Partial least-squares discriminant analysis (PLS-DA) was used as the classification method for modeling the discrimination between normal weight and overweight subjects by visualization of the score plot or *S*-plot using the first and second PLS components. To validate the model, a seven-fold validation was applied to the PLS-DA model, and the reliabilities of the model were further rigorously validated by a permutation test (*n* = 200). The goodness of fit was quantified by *R* ^2^ *Y*, and the predictive ability was quantified by *Q* ^2^ *Y*. Generally, *R* ^2^ *Y* describes how well the data in the training set are mathematically reproduced and varies between 0 and 1 (a value of 1 indicates a model with a perfect fit). Models with *Q* ^2^ *Y* ≥0.5 are considered to have good predictive capabilities.

## Results

### Clinical characteristics, arterial stiffness, and nutrient intake at baseline and 3-year follow-up

There were no significant differences between the control group and the overweight group in baseline characteristics including gender (29 males and 30 females in both groups) and age (control, 46.3±0.71 years; overweight, 46.3±0.73 years). Also, there were no significant changes in smoking and drinking status between baseline and 3-year follow-up in each group (data not shown). At baseline, the overweight group showed higher BMI, waist circumference, triglycerides, FFA, glucose, insulin, HOMA-IR, and hs-CRP, and lower HDL-cholesterol than controls (Table 1). Similarly, after 3 years the overweight group showed higher BMI, waist circumference, systolic and diastolic blood pressure (SBP and DBP), triglyceride, FFA, glucose, insulin, HOMA-IR, and ba-PWV, and lower HDL-cholesterol than controls. Moreover, at the 3-year follow-up the overweight group showed an increase in waist circumference, SBP and DBP, FFA, insulin, HOMA-IR, and ba-PWV compared with baseline levels, and the changes in waist circumference, SBP, DBP, triglyceride, FFA, glucose, insulin, HOMA-IR, hs-CRP, and ba-PWV observed in the overweight group were significantly different from those in the normal weight group after adjusting for baseline levels (Table 1). The estimated total energy intakes for the control and overweight groups were 2,119±41 kcal/d and 2,272±39 kcal/d respectively at baseline (*P* = 0.008) and 2,125±38 kcal/d and 2,283±37 kcal/d at the 3-year follow-up (*P* = 0.003). There were no statistically significant differences in the percentage of total energy intake obtained from macronutrients between baseline and the 3-year follow-up; in particular, the polyunsaturated/monounsaturated/saturated (P/M/S) fat-intake ratio at baseline (control, 1/0.85/0.71; overweight, 1/0.87/0.77) was not significantly different from that at the 3-year follow-up (control, 1/0.82/0.59; overweight, 1/0.89/0.60). There were no significant differences in total energy expenditure or the proportion of subjects who smoke and/or drink alcohol between the baseline and 3-year follow-up data (data not shown).

**Table 1:** Clinical characteristics and brachial-ankle pulse wave velocity at baseline and at the 3-year follow-up.

	Control (*n* = 59)		Overweight (*n* = 59)		*P* ^a^	*P* ^b^	*P* ^c^	*P* ^d^
	Baseline	Follow-up	Baseline	Follow-up				
Body mass index (kg/m^2^)	21.8±0.21	22.3±0.43	27.0±0.20	27.9±0.54	<0.001	<0.001		
Waist (cm)	80.5±0.79	80.9±0.81	86.8±0.73	90.3±0.79***	<0.001	<0.001		
Change	0.37±0.80		3.42±0.79				0.008	<0.001
Waist to hip ratio	0.89±0.01	0.88±0.01	0.91±0.01	0.93±0.01**	0.040	<0.001		
Change	-0.01±0.01		0.02±0.01				0.010	<0.001
Systolic BP (mmHg)	115.9±1.70	116.3±1.93	119.5±1.68	125.1±1.79**	0.138	0.001		
Change	0.34±1.50		5.59±1.70				0.022	0.003
Diastolic BP (mmHg)	71.5±1.44	71.5±1.52	73.4±1.18	76.5±1.38**	0.311	0.016		
Change	0.07±1.34		3.17±1.15				0.081	0.026
Triglyceride (mg/dL)^*∮*^	87.5±6.26	83.9±6.07	141.3±10.3	172.1±12.7	<0.001	<0.001		
Change	-3.66±4.60		30.8±7.68				<0.001	<0.001
Total-cholesterol (mg/dL)^*∮*^	190.6±4.63	189.1±4.08	191.3±4.52	198.3±4.94	0.914	0.189		
LDL-cholesterol (mg/dL)^*∮*^	117.6±4.53	119.1±3.83	117.6±4.32	118.5±4.91	0.985	0.602		
HDL-cholesterol (mg/dL)^*∮*^	55.5±1.99	53.3±2.09	45.4±1.54	45.4±1.43	<0.001	0.005		
Free fatty acid (uEq/L)^*∮*^	555.6±39.2	508.8±28.5	624.1±34.7	824.7±39.6***	0.039	<0.001		
Change	-46.9±41.5		200.6±30.0				<0.001	<0.001
Glucose (mg/dL)^*∮*^	89.8±1.23	89.8±0.97	95.4±1.29	97.0±1.54	0.002	<0.001		
Change	-0.07±1.28		1.62±1.08				0.315	0.010
Insulin (uIU/mL)^*∮*^	8.19±0.42	7.92±0.32	10.0±0.53	11.6±0.64**	0.004	<0.001		
Change	-0.27±0.39		1.41±0.42				0.004	<0.001
HOMA-IR^*∮*^	1.85±0.11	1.77±0.08	2.38±0.14	2.79±0.17**	0.001	<0.001		
Change	-0.08±0.10		0.37±0.11				0.004	<0.001
hs-CRP (mg/dL)^*∮*^	1.03±0.30	0.67±0.10	1.14±0.17	1.66±0.54	0.020	0.080		
Change	-0.35±0.31		0.56±0.58				0.161	0.016
baPWV (cm/sec)^*∮*^	2560.6±46.1	2514.5±47.0	2608.1±43.3	2735.1±57.2***	0.421	0.004		
Change	-46.1±24.7		127.0±29.7				<0.001	<0.001

Mean ±SE.

^∮^tested by logarithmic transformation, *P* ^*a*^-values derived from independent *t*-test in baseline. *P* ^*b*^-values derived from independent *t*-test in follow-up. *P* ^*c*^-values derived from independent *t*-test in change value. *P* ^*d*^-values derived from independent *t*-test in change value after adjustment for baseline.

*P<0.05,

**P<0.01,

***P<0.001 derived from paired *t*-test.

### Plasma metabolic profiling using UPLC-LTQ-Orbitrap mass spectrometry

#### Non-targeted metabolic pattern analysis

**Fig 1:**
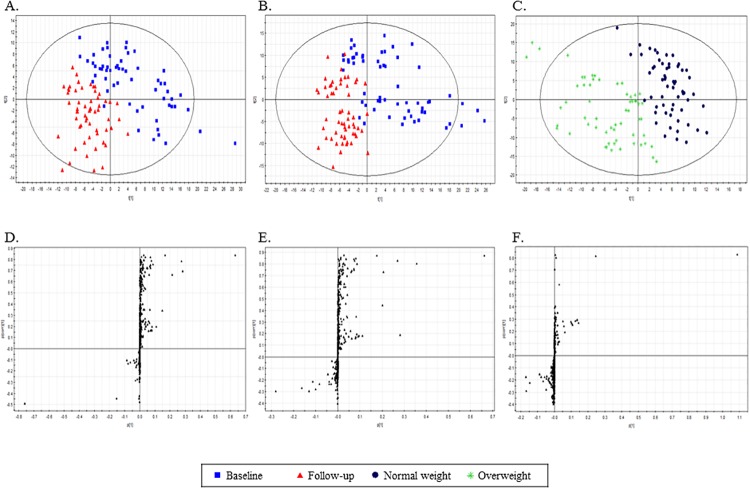
Identification of plasma metabolites that were significantly altered at the 3-year follow up. A: Score plots from PLS-DA models for normal weight subjects (*n* = 59) at baseline and at 3-year follow-up. B: Score plots from PLS-DA models for overweight subjects (*n* = 59) at baseline and at 3-year follow-up. C: Score plots from PLS-DA models for normal weight and overweight subjects at 3-year follow-up. D, E, F: S-plots for covariance [p] and reliability correlation [p(corr)] from PLS-DA models.

**Fig 2:**
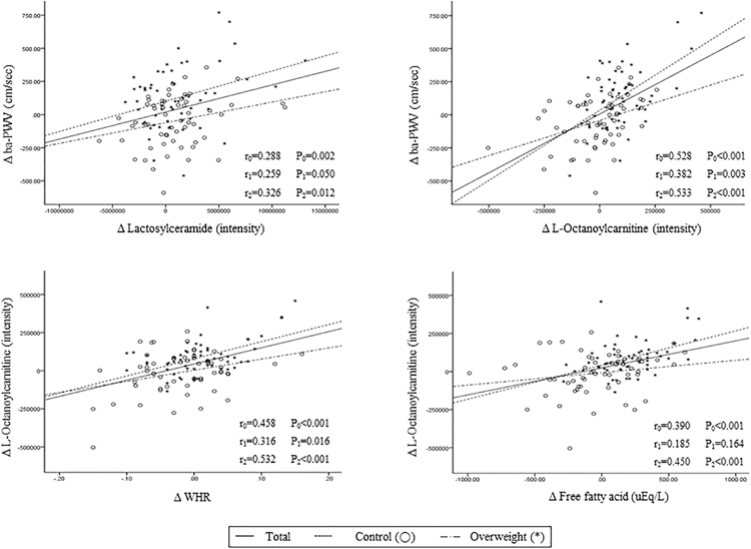
Correlations between changes in ba-PWV, lactosylceramide, L-Octanoylcarnitine, free fatty acids, and waist to hip ratio between baseline and 3-year follow-up in middle-aged subjects. Tested by Pearson correlation. r_0_: correlation coefficient in total population, r_1_: correlation coefficient in control group, r_2_: correlation coefficient in overweight group.

The mass spectrometry (MS) data of plasma metabolites obtained at baseline and the 3-year follow-up were analyzed with a PLS-DA score plot. PLS-DAs were conducted for the following three combinations of groups: (1) normal weight at baseline and normal weight at 3-year follow-up (Fig. 1A); (2) overweight at baseline and overweight at 3-year follow-up (Fig. 1B); and (3) normal weight and overweight at 3-year follow-up (Fig. 1C). The two-component PLS-DA scatter plots of the plasma metabolites showed distinct clustering and clear separation for subjects with normal weight at baseline and normal weight at 3-year follow-up [with R^2^X(cum) = 0.155, R^2^Y(cum) = 0.695, *Q* ^*2*^ *Y*(cum) = 0.502] (Fig. 1A). The PLS-DA model was validated using a permutation test, which indicated an *R* ^2^ *Y* intercept value of 0.0762 and a *Q* ^2^ *Y* intercept value of —0.158. Similarly, the two-component PLS-DA scatter plots of the plasma metabolites showed separation for subjects with overweight baseline and overweight 3-year follow-up [with R^2^X(cum) = 0.203, R^2^Y(cum) = 0.653, *Q* ^*2*^ *Y*(cum) = 0.537] (Fig. 1B) and a permutation test indicated an *R* ^2^ *Y* intercept value of 0.0642 and a *Q* ^2^ *Y* intercept value of —0.176. Additionally, the two-component PLS-DA scatter plots of the plasma metabolites showed clear separation for subjects with normal weight 3-year follow-up and overweight 3-year follow-up [with R^2^X(cum) = 0.197, R^2^Y(cum) = 0.744, *Q* ^*2*^ *Y*(cum) = 0.644] (Fig. 1C) and a permutation test indicated an *R* ^2^ *Y* intercept value of 0.0884 and a *Q* ^2^ *Y* intercept value of -*0*.*152*. To identify the metabolites that differentially determined the data at baseline and 3-year follow-up, *S*-plots of p(1) and p(corr)(1) were generated using centroid scaling (Fig. 1D, E and F). The *S*-plots revealed that metabolites with higher or lower p(corr) values were more relevant for discriminating between the two groups.

#### Identification of plasma metabolites

Among 764 metabolites in plasma, the metabolites (variables) that played important roles in the separation between the groups were selected according to the Variable Important in the Projection (VIP) parameter, with VIP values >1.0 indicating a high relevance for the difference between the sample groups. Seventy-six metabolites were selected based on VIP values >1.0; of these 23 metabolites were identified and 53 were unknown. There were no significant differences in baseline metabolites between control and overweight groups. After the 3-year follow-up, the control group showed significant decreases in the following 12 metabolites: lysophosphatidylcholines (lysoPCs) containing C14:0, C16:1, C16:0, C17:0, C18:1, C18:0, C20:4, C20:3, and C22:6 (Suppl. 1), PC (16:0/18:1), PC (18:0/18:2), and PC (18:0/20:4) (Table 2). The overweight group showed significant decreases in 11 metabolites—lysoPCs containing C14:0, C16:1, C16:0, C17:0, C18:2, C18:1, C18:0, C20:4, C20:3, and C22:6 (Suppl. 1), and PC (18:0/20:4) (Table 2)—and significant increases in 6 metabolites—L-leucine, palmitic amide, L-octanoylcarnitine, decanoylcarnitine, PC (18:2/18:2), and lactosylceramide (Table 2). Next, we compared changes in metabolites (differences between baseline and 3-year follow-up) between the control and overweight groups. The overweight group showed greater increases in L-octanoylcarnitine (*q* = 0.006) and decanoylcarnitine (*q* = 0.007) than the control group (Table 2) and higher peak intensities of L-leucine, L-octanoylcarnitine, and decanoylcarnitine at the 3-year follow-up compared with the control group (Table 2). The level of L-octanoylcarnitine at 3-year follow-up was significantly higher in overweight group, when the subjects were divided according to sex or menopause status (*q* = 0.002 in male, *q* = 0.001 in female; *q* = 0.012 in pre-menopause, *q* = 0.017 in post-menopause, respectively).

**Table 2:** Identification of plasma metabolites at baseline and 3-year follow-up in control and overweight individuals.

Identity	Formula [M +H]^+^	Exact Mass (M+H)	Normalized peak intensities				VIP		
			Control (n = 59)		Overweight (n = 59)		Baseline vs. Follow-up		3-year
			Baseline	Follow-up	Baseline	Follow-up	Control	Overwt	Controlvs.Overwt
L-Valine	C_5_H_11_NO_2_	118.0868	1524590±43596	1467528±41252	1617763±47873	1619518±43944	0.3414	0.0712	1.4575
L-Leucine	C_6_H_13_NO_2_	132.1025	5362065±182114	5394995±132472	5565474±159151	6169537±157030** ^,^ ^*†*^	1.8500	1.1943	7.8558
L-Phenylalanine	C_9_H_11_NO_2_	166.0868	3636600±131353	3579944±135114	3755242±159444	3827672±190594	0.7550	0.8943	2.1233
Palmitic amide	C_16_H_33_NO	256.2640	469819±55788	697467±80228	483560±53617	735686±79233*	1.6853	1.4459	0.4678
Oleamide	C_18_H_35_NO	282.2797	3924070±363635	4954137±416864	4244742±412439	5177850±391846	7.8561	5.6326	2.4200
L-Octanoylcarnitine	C_15_H_29_NO_4_	288.2175	240163±19550	232019±17052	260632±14980	348114±18898***, ^*††*^	0.0604	0.4405	1.1628
Change			-10394±18032		87483±14961^*‡‡*^				
Decanoylcarnitine	C_17_H_33_NO_4_	316.2488	366576±25936	344046±22815	411930±22798	521371±25727***, ^*††*^	0.1298	0.5994	1.8195
Change			-22531±23778		109441±21029^*‡‡*^				
PC (16:0/18:2)	C_42_H_80_NO_8_P	758.5700	4805090±272599	5004712±395273	4770408±267562	5308029±322755	4.8455	6.7249	5.5254
PC (16:0/18:1)	C_42_H_82_NO_8_P	760.5856	1642078±108170	1184513±60033***	1450047±78254	1308776±66476	2.9894	0.6693	1.3506
PC (18:2/18:2)	C_44_H_80_NO_8_P	782.5700	3545437±146248	3839923±168360	3527916±162629	4443089±223941**	1.8499	4.5592	6.0670
PC (18:0/18:2)	C_44_H_84_NO_8_P	786.6013	1120555±85867	852204±56389*	967549±91416	819526±43182	1.6556	0.8123	0.5889
Lactosylceramide (d18:1/12:0)	C_42_H_79_NO_13_	806.5630	4210199±312480	5038795±289619	4676478±232891	5833895±410936*	5.4776	5.5056	6.3984
PC (18:0/20:4)	C_46_H_84_NO_8_P	810.6013	1491813±81564	1210223±74248**	1523732±99005	1183146±62603**	2.0140	1.8165	0.3348

Mean ±SE.

*q<0.05,

**q<0.01,

***q<0.001 derived from paired *t*-test.

^*†*^ *q*<0.05,

^*††*^ *q*<0.01,

^*†††*^ *q*<0.001 derived from independent *t*-test in follow-up.

^*‡*^ *q*<0.05,

^*‡‡*^ *q*<0.01,

^*‡‡‡*^ *q*<0.001 derived from changed values between control and overweight group. VIP, Variable Important in the Projection.

### Changes in brachial-ankle pulse wave velocity correlate with changes in biochemical parameters and major plasma metabolites

In all subjects (*n* = 118), changes (△) in ba-PWV positively correlated with △ waist circumference (*r* = 0.294, *P* = 0.001), △ WHR (*r* = 0.268, *P* = 0.003), △ SBP (*r* = 0.319, *P<*0.001), △ triglyceride (*r* = 0.214, *P* = 0.020), △ hs-CRP (*r* = 0.233, *P* = 0.012), △ L-octanoylcarnitine (*r* = 0.528, *P*<0.001), △ decanoylcarnitine (*r* = 0.452, *P*<0.001), and △ lactosylceramide (*r* = 0.288, *P* = 0.002) (Fig. 2). On the basis of these results, we performed a multiple regression analysis to determine independent predictors of △ ba-PWV. Age, gender, baseline BMI, △ waist circumference, △ SBP, △ triglyceride, △ HOMA-IR, △ insulin, △ hs-CRP, △ L-leucine, △ L-octanoylcarnitine, △ decanoylcarnitine, and △ lactosylceramide were tested. Changes in L-octanoylcarnitine (standardized *β* = 0.428, *P* = 0.003) and lactosylceramide (standardized *β* = 0.201, *P* = 0.012) were the strongest independent predictors of △ ba-PWV, although baseline BMI (standardized *β* = 0.185, *P* = 0.034) and △ SBP (standardized *β* = 0.196, *P* = 0.020) were also significant.

### Changes in L-octanoylcarnitine and lactosylceramide correlate with changes in biochemical parameters and other major plasma metabolites

Changes in L-octanoylcarnitine positively correlated with △ WHR (Fig. 2), △ total cholesterol, △ FFA (Fig. 2), △ L-leucine, △ L-phenylalanine, and △ decanoylcarnitine (*r* = 0.815, *P*<0.001). Changes in lactosylceramide correlated positively with △ decanoylcarnitine and negatively with △ L-valine, △ lysoPCs (14:0, 16:0, 18:2, 18:0, 20:3, and 22:6), and △ PC (18:0/20:4) (*r* = 0.266, *P* = 0.004). Additionally, △ L-valine (*r* = 0.411, *P*<0.001) and △ L-leucine (*r* = 0.479, *P*<0.001) positively correlated with △ HOMA-IR.

## Discussion

The results of this study revealed that middle-aged overweight subjects showed a substantial increase in ba-PWV, as an index of central arterial stiffness [18,19] during the follow-up period that exceeded that observed in the normal weight controls. Additionally, changes in L-octanoylcarnitine, a physiologically active form of octanoylcarnitine, and lactosylceramide emerged as independent predictors of ba-PWV change, as did baseline BMI and changes in SBP. Moreover, mean changes in both ba-PWV and SBP, markers of arterial stiffness, positively correlated with changes in L-octanoylcarnitine and lactosylceramide. This finding suggests that the duration of overweight is an important risk factor for increased arterial stiffness, especially during middle age, and that this increase in arterial stiffness might be associated with age-related increases in plasma L-octanoylcarnitine and lactosylceramide.

In addition to the increase in ba-PWV, the overweight subjects showed a 34% increase in L-octanoylcarnitine regardless of sex or menopause status, and a 27% increase in decanoylcarnitine over the 3-year period, whereas no significant changes occurred in normal weight subjects. Acylcarnitines are carnitine esters derived from fatty acids or amino acids that are transferred into the mitochondria [20]. Elevated acylcarnitine production can occur when β-oxidation rates are in excess of complete oxidation to carbon dioxide through the tricarboxylic acid cycle [20]. Thus, medium-chain acylcarnitines including octanoylcarnitine and decanoylcarnitine are known to be intermediate fatty acid β-oxidation byproducts [21] and markers for incomplete fatty acid oxidation [22]. Indeed, higher body fat has been shown to correlate with overloaded β-oxidation of fatty acids, which leads predominantly to an increased amount of medium-chain acylcarnitines [22]. Obese adults with or without type 2 diabetes are characterized by dysregulated fatty acid and amino acid metabolism [23]. Recently demonstrated elevations in acylcarnitine and amino acid concentrations in obese compared with lean [24,25]. Chronic overfeeding leads to increased adiposity, hyperlipidemia, and ectopic accumulation of triglyceride and other lipid-derived metabolites in tissues such as liver, pancreas, skeletal muscle, and heart [26,27]. Seiler et al. [28] found that muscle levels of medium chain acyl-CoAs, along with several even chain acylcarnitine intermediates of fat oxidation, were elevated in rodent models of obesity. Therefore, the increase in octanoylcarnitine and decanoylcarnitine in the middle-aged overweight subjects of this study could be related to upregulated β-oxidation of fatty acids, possibly due to a higher load of FFA or higher abdominal fat accumulation [22,29]. The changes in octanoylcarnitine and decanoylcarnitine in the present study appeared to be closely associated with changes in fasting FFA and WHR. The waist circumference in overweight subjects showed significant increase after 3-year follow-up (86.8±0.73 cm vs. 90.3±0.79 cm), also, free fatty acid level (624.1±34.7 uEq/L vs. 824.7±39.6 uEq/L). However, acylcarnitine concentrations increase in the plasma of lean, insulinsensitive subjects during long-term fasting [30] and relatively healthy overweight subjects during caloric restriction [31]. These observations suggest that the acylcarnitine increase in obesity may not only be due to an impairment of metabolism but also be a natural response to an excess supply of lipid. Thus, increased production of acylcarnitine could result from excess fatty acid flux emanating from lipid stored either intracellularly or peripherally. Indeed, plasma acylcarnitine concentrations are reduced during an oral glucose tolerance test and during a euglycemic-hyperinsulinemic clamp, both procedures that reduce plasma FFA concentrations significantly [32,33]. Yet, under these conditions, obese and diabetic subjects maintained higher acylcarnitine concentrations than lean controls [32]. As a result of a growing body of data in the literature, acylcarnitine levels, additionally, are proposed as biomarkers in subjects with insulin resistance [32,34]. Indeed, acylcarnitine concentrations are higher in the fasting state in insulin-resistant populations and decrease less during glucose challenge tests [32,33]. This latter observation may be due to a continued release of adipose FFA as a result of adipose insulin resistance or could be due to use of intramyocellular lipid [35]. In present study, HOMA-IR, L-octanoylcarnitine, and decanoylcarnitine were increased in overweight group after 3-year follow-up, which are coincide with previous studies.

White adipose tissue has become appreciated as a potential player in whole-body metabolism of branched-chain amino acids (BCAAs) [36,37]. The mRNA abundance of BCAA catabolic enzymes has been reported to be markedly reduced in omental (but not subcutaneous) white adipose tissue of obese individuals with metabolic syndrome compared with weight-matched healthy obese subjects, raising the possibility that visceral adipose tissue contributes to the BCAA metabolic phenotype of metabolically compromised individuals [36]. In this study, the overweight subjects showed a significant increase of 3.42 cm in waist circumference over the 3-year period and higher plasma leucine/isoleucine levels compared with normal weight individuals at the 3-year follow-up. It has been suggested that adiposity signals *per se* downregulate white adipose tissue BCAA enzyme expression and that an increase in systemic BCAAs tracks the degree of insulin resistance, worsening blood sugar control [36]. Many studies reported the correlation between insulin resistance and arterial stiffness. In the cross-sectional analysis of ARIC (Atherosclerosis Risk in Communities), arteries appear to become stiffer at increasing concentrations of fasting glucose and insulin, independent of race or gender [38]. In 2,488 adults participating in the Health ABC study, increased serum insulin concentrations and visceral fat volume measured with computed tomography were associated with increased aortic stiffness measured by PWV [39]. Increased BCAAs may increase activation of mTOR/S6K1 kinase pathway, which phosphorylates insulin receptor substrate 1 (IRS-1), with a subsequent decline in IRS-1 associated phosphatidylinositide 3-kinase (PI 3-kinase) activity. Reduced PI 3-kinase activity may result in diminished glucose uptake and glucose utilization [40]. Decreased glucose transport is sensed at the pancreatic β-cells, and may result in a compensatory increase in insulin secretion. Increased insulin secretion may lead to an excessive growth promoting signal, which stimulates various pro-atherogenic events in vascular smooth muscle and endothelial cells [41], which may be related to arterial stiffness. Indeed, the strongly positive correlations among valine, leucine, and HOMA-IR in this study are consistent with previous findings [24]. Thus, the greater increases in systolic and diastolic blood pressure, triglyceride, and HOMA-IR index in the overweight group compared with normal weight subjects could support the previous report of a close association between systemic BCAAs and an insulin resistance phenotype, and a subsequent arterial stiffness [42].

In this study, age-related decreases in all lysoPCs were found in both normal weight and overweight groups. This result is in agreement with previous animal studies, which showed a decrease in lyosPCs with advancing age [43]. Additionally, age-related increases in plasma palmitic amide and lactosylceramide were also observed in overweight subjects. Lactosylceramide, one of the ubiquitous glycosphingolipids, is generated in endothelial cells after treatment with vascular endothelial growth factor, which has been implicated in vascular pathologies [44]. Similarly, a change in lactosylceramide was one of the independent predictors of ba-PWV change in this study. In addition, the negative association between lactosylceramide and PC (18:0/20:4) in the current study supports previous findings that lactosylceramide stimulates the activity of cytosolic Ca^2+^-dependent PLA_2_ [45], of which the preferred substrates are phospholipids containing arachidonic acid (20:4 ω6) at the *sn*-2 site [46].

Adipose tissue affects to metabolism by releasing non-esterified fatty acids and glycerol, hormones including leptin and adiponectin, and proinflammatory cytokines [47]. The release of non-esterified fatty acids may be the most important factor in modulating insulin sensitivity, and increased levels of non-esterified fatty acids are associated with the insulin resistance observed in obesity and type 2 diabetes [47]. In addition to adipocyte-derived factors, increased release of TNF-α, IL-6, monocyte chemo-attractant protein-1 (MCP-1) and additional products of macrophages and other cells that present in adipose tissue might also have a role in the development of insulin resistance [48]. In the present study, the overweight group showed glucose, insulin, HOMA-IR, and hs-CRP than control group, indicating insulin resistance and increased inflammatory status than controls. Therefore, a significant change in metabolites in overweight group without lifestyle changes might be triggered by adipocyte-derived factors that may elicited insulin resistance and related inflammatory processes.

Although a large number of metabolite markers were detected by UPLC-LTQ-Orbitrap MS in this study, most of these metabolites are currently unidentified. Large databases of endogenous biomolecules have not yet been constructed for use with LC-MS-based techniques for metabolomics research [49]. The relatively small sample size in this study may not be sufficiently large to detect all overweight-associated metabolic changes and arterial stiffness. Despite this limitation, our approach using UPLC-LTQ-Orbitrap MS-based metabolomics and multivariate data analysis showed greater increases in L-octanoylcarnitine and decanoylcarnitine in the overweight group compared with the control group. Additionally, the overweight group exhibited increases in L-leucine, lacosylceramide, and ba-PWV over a 3-year period. Furthermore, changes in L-octanoylcarnitine and lactosylceramide independently predicted ba-PWV changes, as did baseline BMI and changes in SBP. This finding suggests that the duration of overweight is an important aggravating factor for arterial stiffness, especially during middle age, and that this increased arterial stiffness might be associated with an age-related increase in plasma L-octanoylcarnitine and lactosylceramide.

## Conclusions

In summary, our results suggest that increased arterial stiffness and metabolic dysfunction associated with abdominal fat accumulation occur in middle-aged overweight individuals. Further studies are needed to determine whether these changes are modifiable by weight loss.

## Supporting Information

S1 Table Supporting Table. (DOCX)
