# Supplementary material for: Association between Arterial Stiffness and Serum L-Octanoylcarnitine and Lactosylceramide in Overweight Middle-Aged Subjects: 3-Year Follow-Up Study
Source: PLoS One. 2015 Mar 17;10(3):e0119519. doi: 10.1371/journal.pone.0119519 (PMC4363527; doi:10.1371/journal.pone.0119519)
Supplement: S1 Table — (DOCX) [file pone.0119519.s001.docx]

**S1 Table. Identification of plasma lysophosphatidylcholines at baseline and 3-year follow-up in control and overweight individuals**

| **Identity** | **Formula**  **[M +H]^+^** | **Exact**  **Mass**  **(M+H)** |  | **Normalized peak intensities** | | | | | | | | |  | **VIP** | | | |
| --- | --- | --- | --- | --- | --- | --- | --- | --- | --- | --- | --- | --- | --- | --- | --- | --- | --- |
|  |  |  |  | **Control (n=59)** | | | |  | **Overweight (n=59)** | | | |  | **Baseline vs. Follow-up** | |  | **3-year** |
|  |  |  |  | **Baseline** | | **Follow-up** | |  | **Baseline** | | **Follow-up** | |  | **Control** | **Overwt** |  | **Control**  **vs.**  **Overwt** |
| LysoPC (14:0) | C_22_H_46_NO_7_P | 468.3090 |  | 496246 | ±34135 | 368612 | ±19487*^**^* |  | 592857 | ±39782 | 372452 | ±17324*^***^* |  | 0.7448 | 1.0275 |  | 0.0419 |
| LysoPC (16:1) | C_24_H_48_NO_7_P | 494.3247 |  | 1184518 | ±76837 | 796158 | ±41323*^***^* |  | 1226169 | ±69536 | 789994 | ±34586*^***^* |  | 2.2520 | 2.0446 |  | 0.1911 |
| LysoPC (16:0) | C_24_H_50_NO_7_P | 496.3403 |  | 13890520 | ±495190 | 11154737 | ±268707*^***^* |  | 14582484 | ±467476 | 11000191 | ±198638*^***^* |  | 15.8544 | 16.6789 |  | 1.9314 |
| LysoPC (17:0) | C_25_H_52_NO_7_P | 510.3560 |  | 765578 | ±47478 | 515635 | ±24684*^***^* |  | 814617 | ±56260 | 484629 | ±23645*^***^* |  | 1.4561 | 1.5396 |  | 0.3313 |
| LysoPC (18:2) | C_26_H_50_NO_7_P | 520.3403 |  | 5040139 | ±211025 | 4601299 | ±153221 |  | 5096388 | ±176350 | 4115088 | ±106848*^***^* |  | 3.3552 | 4.6314 |  | 5.6863 |
| LysoPC (18:1) | C_26_H_52_NO_7_P | 522.3560 |  | 5161739 | ±232027 | 4153155 | ±140342*^***^* |  | 5254377 | ±201104 | 3892501 | ±108804*^***^* |  | 5.8897 | 6.3730 |  | 3.3060 |
| LysoPC (18:0) | C_26_H_54_NO_7_P | 524.3716 |  | 6405156 | ±270611 | 5299323 | ±167016*^**^* |  | 7015335 | ±265016 | 4948060 | ±135665*^***^* |  | 6.4232 | 9.6272 |  | 3.6114 |
| LysoPC (20:4) | C_28_H_50_NO_7_P | 544.3403 |  | 1487192 | ±61853 | 1184837 | ±37234*^***^* |  | 1551956 | ±58441 | 1176628 | ±35039*^***^* |  | 1.7520 | 1.7521 |  | 0.1886 |
| LysoPC (20:3) | C_28_H_52_NO_7_P | 546.3560 |  | 680240 | ±43731 | 520606 | ±26609*^**^* |  | 730498 | ±36396 | 484516 | ±19835*^***^* |  | 0.9438 | 1.1451 |  | 0.3774 |
| LysoPC (22:6) | C_30_H_50_NO_7_P | 568.3403 |  | 1060023 | ±65453 | 755852 | ±34660*^***^* |  | 1076408 | ±65702 | 736513 | ±28629*^***^* |  | 1.7628 | 1.6133 |  | 0.2758 |

Mean ± SE. *^*^q* <0.05, *^**^q* <0.01, *^***^q* <0.001 derived from paired *t*-test. VIP, Variable Important in the Projection.
